# Neural correlates of individual differences in manual imitation fidelity

**DOI:** 10.3389/fnint.2012.00091

**Published:** 2012-10-16

**Authors:** Lieke Braadbaart, Gordon D. Waiter, Justin H. G. Williams

**Affiliations:** ^1^School of Psychology, University of AberdeenAberdeen, UK; ^2^Aberdeen Biomedical Imaging Centre, Institute of Medical Sciences, University of AberdeenAberdeen, UK; ^3^School of Medicine and Dentistry, Clinical Research Centre, University of Aberdeen, Royal Cornhill HospitalAberdeen, UK

**Keywords:** manual imitation, fMRI BOLD, mirror neuron areas, kinematics, correlated activity

## Abstract

Imitation is crucial for social learning, and so it is important to identify what determines between-subject variability in imitation fidelity. This might help explain what makes some people, like those with social difficulties such as in autism spectrum disorder (ASD), significantly worse at performance on these tasks than others. A novel paradigm was developed to provide objective measures of imitation fidelity in which participants used a touchscreen to imitate videos of a model drawing different shapes. Comparisons between model and participants' kinematic data provided three measures of imitative fidelity. We hypothesized that imitative ability would predict variation in BOLD signal whilst performing a simple imitation task in the MRI-scanner. In particular, an overall measure of accuracy (correlation between model and imitator) would predict activity in the overarching imitation system, whereas bias would be subject to more general aspects of motor control. Participants lying in the MRI-scanner were instructed to imitate different grips on a handle, or to watch someone or a circle moving the handle. Our hypothesis was partly confirmed as correlation between model and imitator was mediated by somatosensory cortex but also ventromedial prefrontal cortex, and bias was mediated mainly by cerebellum but also by the medial frontal and parietal cortices and insula. We suggest that this variance differentially reflects cognitive functions such as feedback-sensitivity and reward-dependent learning, contributing significantly to variability in individuals' imitative abilities as characterized by objective kinematic measures.

## Introduction

The ability to imitate, defined as the ability to learn how to do something by watching how someone else does it, is arguably the characteristic that best differentiates human cognition from other animals (Whiten, 2006). While studies have been increasingly demonstrating the capacity for imitation in non-human primates in the last 10 years (Whiten and van Schaik, 2007), the breadth of human ability far outweighs that seen in other animals. It would seem that the evolution of our capacity for imitation is what has provided us as a species with the rich cultural diversity that we take for granted. It is also argued that the capacity for imitation, which requires the ability to detect similarities between the observer and the observed, is closely linked to the capacity for “identification” with others (Hobson and Meyer, 2006), social cognition (Uddin et al., 2007), empathy (Sommerville and Decety, 2006) and the simulation theory of mind which allows a person to understand another's mental state by imagining themselves in their position (Meltzoff and Gopnik, 1993; Gallese and Goldman, 1998; Gallese, 2003; Hurley and Chater, 2005). Research into how imitation works becomes even more important when looking at people who do not possess the ability to put themselves in another's shoes, figuratively speaking. The most prominent group of people who struggle with social and imitation deficits are those with autism spectrum disorder (ASD; e.g., Rogers and Williams, 2006). Understanding the neural basis for the capacity to detect and develop the correspondences between observations of others' behavior and one's own coding for that same behavior may be essential to understanding social cognition and related deficits in disorders such as ASD.

Research in the area of imitation over the last decade has been dominated by the hypothesis that a single, “direct-matching” mechanism exists that couples neural codings for observation to neural codings for the same action, and that this takes the form of a “mirror neuron” system (Iacoboni et al., 1999; Rizzolatti and Craighero, 2004). Mirror neurons fire not only when executing an action, but also when observing that same action, and therefore offer a potential cross-modal mapping function, so that the observation of others' actions enables the observer to experience them as if performing them him- or herself. In the macaque, mirror neurons have been located in the inferior parietal and ventral premotor cortices (Gallese et al., 1996; Fogassi et al., 2005). There is evidence from functional magnetic resonance imaging (fMRI) and electrophysiological methods for the existence of a putative mirror neuron system in humans (Iacoboni and Dapretto, 2006; Chong et al., 2008). A recent meta-analysis of imitation (Caspers et al., 2010) identified a number of brain areas as being commonly activated across a range of imitation studies supporting the idea of a widespread imitation system. Areas included the inferior frontal gyrus (Broca's area), the inferior parietal lobe, somatosensory cortex, premotor cortex, and fusiform gyrus. In imitation learning it has been suggested that a similar mechanism is utilized to compare others' actions with one's own (Oztop and Arbib, 2002), and that a deficit in the mirror mechanism is responsible for the social deficits found in ASD (Iacoboni and Dapretto, 2006; Williams et al., 2006).

In addition to action-perception matching, imitation may also include reinforcement learning and motor control. Learning an action and motor control both rely on the interplay between sensory feedback and motor command execution. Imitation takes this cross-modal action translation one step further, because the sensory signals which normally come from our own body are instead created by another person. Without these self-induced signals, the brain has to compensate in order to accurately reflect the actions of another person, relying on visuospatial and auditory information and our own motor system to fill in the sensory gaps (Wolpert et al., 2003). It follows from this that previously learned actions are easier to imitate than novel actions as they correspond to well-established sensory-motor loops. Furthermore, as imitation depends upon different processes, including action perception, cross-modal matching and motor control, a broad system of brain activity common to all imitation is required. A separate question then arises as to how the various components of an imitation system might contribute to imitative performance. The deconstruction of imitation has previously been investigated in a study on hand gestures by Gold et al. (2008). They used a data-glove to track spatiotemporal motions as the participants imitated different gesture sequences. Gold and colleagues found that various measures of error related to different components of the imitative action, and that these measures managed to differentiate between effects of spatial memory and complexity. This suggests that the overarching imitation system might not be at fault when a person fails to imitate, but that instead a component of the imitation system could be responsible for the failure. By deconstructing the imitation system, differentiation between the possible causes of imitation deficits will become possible.

If imitative ability predicts social cognitive ability, then understanding the causes of variability in imitative performance becomes important for understanding how social cognition varies within a population. One way of exploring this question is to look at whether a neural system employed for imitation shows variability in function not according to the difficulty of the task but relative to the imitative ability of the participants. Therefore, by contrasting a very simple imitation task with a more difficult one, the underlying collective imitation brain mechanism would vary in its level of activation according to the efficiency of the imitation system. We would expect that the better a person is at imitating, the easier they would find the simple fMRI task and less blood oxygen level dependent (BOLD) activity would then be associated with the task. We recently designed a behavioral task that provides a quantitative measure of imitation ability using custom-built software (Culmer et al., 2009) to derive the kinematic parameters of actions, which can then be directly compared with the kinematics of the model's actions. For the purpose of this study, we considered path length (which corresponds to size of action) and path speed (which corresponds to how fast the action is executed). If this is done for a series of actions, several measures of imitation ability can be derived.

### Correlation

The correlation coefficient provides a measure of degree of dependency between two datasets. If a correlation is perfect between the kinematics of a set of modeled and a set of imitated actions, then the two sets of variables will be completely dependent upon each other and all variability in the imitator's actions will be accounted for by variability in the modeled actions.

### Proportional bias

Even if the value of the correlation coefficient is perfect at 1, there might still be a difference between the absolute values of the model and participant's performance as the imitator may increase speed or size at a slower or faster rate than the model. The slope of the regression line provides information on the relative amount of change between model and imitator across trials and provides a measure of the imitator's inherent bias in drawing the modeled actions.

### Absolute (mean) error

This is the mean amount of difference between the kinematic parameters of model and imitator, irrespective of magnitude of stimulus. It reflects a combination of accuracy and bias.

We hypothesized that these three objective measures would predict activity in neural systems involved in imitation during fMRI of a simple manual imitation task. We also hypothesized that the different measures would correspond to different aspects of these neural systems, which would reflect a variance in vulnerability to the different types of inconsistency. In particular, the dependency measure (correlation coefficient “*R*”) should be the most sensitive to functions controlling the dependency of motor output on sensory input, and would therefore correlate with activity in the action-perception matching system. In contrast, the bias measure (“*m*”) would be most influenced by mechanisms controlling absolute values of motor output and so would reflect more communal motor control functions.

## Methods

### Participants

Sixteen males were recruited to participate from the University of Aberdeen. Their age ranged from 19 to 43, with a mean age of 26.7(SD: 7.19). All participants were right-handed, with no history of illnesses that could affect the brain.

### MRI

MRI data was collected using a 3.0 T scanner (Achieva X-series, Philips Medical, Best, The Netherlands). An eight-channel phased-array head coil was used to obtain high resolution gradient echo 3D volumetric images and a set of functional images using BOLD contrast. The high-resolution images were collected using a T1 weighted sequence with the following parameters: field of view, 24 cm; 20/6, TR/TE; flip angle, 35°; slices, 124; slice thickness, 1.0 mm; matrix, 256 × 256. Functional MR images were acquired in the axial plane with a T2^*^-weighted single shot, gradient-echo, echo-planar pulse sequence with the following parameters: field of view, 24 cm; 2500/30, TR/TE; flip angle, 78°, slices, 30; slice thickness, 5 mm; matrix, 96 × 96. The head was firmly stabilized in the head coil, leaving little room to move.

### Functional imaging task

**Figure 1:**
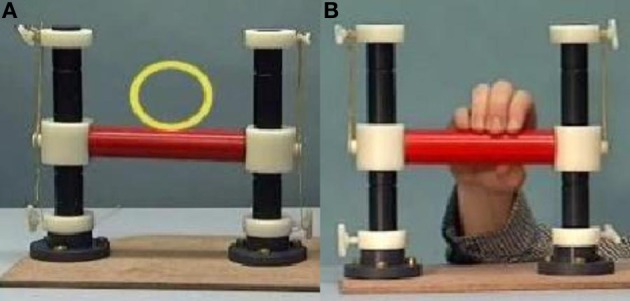
**Video stills of Rest (A) and Move/Watch (B) stimuli**.

Participants were asked to lie in the scanner with a handle by their right side. On a screen they were presented with three conditions using Presentation (version 14). In the first condition, “Rest”, participants were shown a video of the handle moving by itself, with a yellow circle moving with it. In the second condition, “Move”, they were presented with short video clips of a person manipulating the handle and were instructed to imitate these manipulations as they were being shown (see Figure 1). For example, when the participant saw the hand on screen push the handle with only one finger, the participant simultaneously performed the same action. The third condition, “Watch”, showed the same handle manipulations, but this time participants were instructed to observe without moving. Each condition lasted approximately 30 s, consisting of a 5-s instruction screen and six 4-s videos. The three conditions were repeated six times, with a total run-time of 9.5 min. Videos were presented in a pseudo-random order, which was the same for each participant. EEG data was collected simultaneously inside the scanner, to be reported elsewhere.

### Imitation task

**Figure 2:**
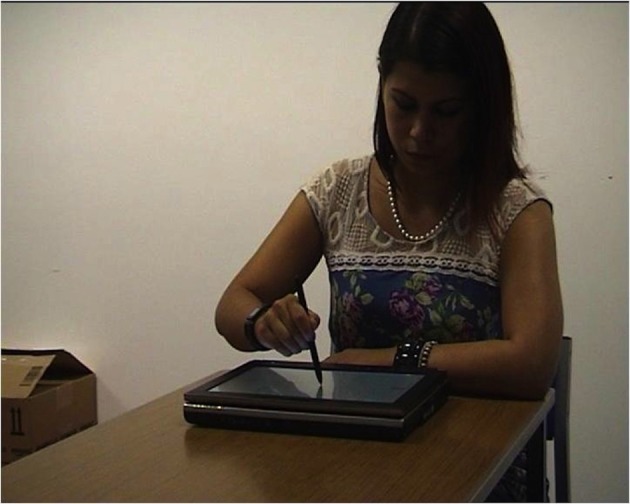
**Still frame of video-clip showing model drawing stimuli**.

A computer was used to assess participants' imitation abilities by exploring how well they imitated drawing actions. Participants watched videos that showed a model tracing a simple shape with pen on the touch-sensitive screen of a portable computer, although the angle of the video was such that the participants could not see the shape on the computer (example in Figure 2). There were five different shapes (circle, oval, square, triangle, and pentagon), drawn at three different speeds (slow, normal, and fast), in three different sizes (small, medium, and large), leading to a total of 45 videos presented in a semi-randomized order, although for one participant only 36 tasks could be analysed due to technical difficulties. After each video, the participant was asked to replicate the drawing they had just seen the model make as closely as possible in size, shape and speed, using the same touch-screen computer with digital pen that the model in the videos used. The position of the pen on the screen was recorded, to be analysed using kinematic assessment tool (KAT) software which automatically generated path length and duration measures for each trial (for a detailed description of how path length and time measures were generated see Culmer et al., 2009). Dividing path length by duration generated a measure of average speed. Measures of imitation accuracy could then be obtained by comparing model and participant parameters. Separate measures of imitation were calculated for path length and average movement speed.

In the first stage of analysis, path length and time measures from the 45 drawing trials of each participant were plotted against those of the model, revealing a correlation between each movement parameter of the participant and that of the model. We considered that the degree of scatter (measured by the strength of the correlation “*R*”) reflected the accuracy of imitation, whereas the gradient of the slope (“*m*” from the regression equation *y* = mx + c), reflects the proportion of change by the imitator across trials as a proportion of the model's change. Mean absolute error between model and participant was also derived through a root mean square error (RMSE) score.

### fMRI analysis

Functional MRI data was analysed using MATLAB software with SPM8 (http://www.fil.ion.ucl.ac.uk/spm/software/spm8/). The 220 functional images were realigned to the first image, whereby a maximum translation and rotation of 1.5 mm/degrees was maintained for all but two participants (with acceptable transgressions of 2.5 mm and −5°). The structural scans were then co-registered to a mean generated from all functional scans, after which they were segmented. All scans were normalized to the standard SPM MNI template, after which the functional scans were smoothed with an 8 mm FWHM Gaussian kernel, completing the pre-processing. The smoothed images were modeled using a general linear model according to the condition blocks, using the movement data from realignment as a regressor. Two-sample *t*-tests generated Move-greater-than-Rest (“Imitate”) and Watch-greater-than-Rest (“Observe”) BOLD contrasts for each individual.

The individual Imitate and Observe contrasts were used in multiple regression analyses with correlation, RMSE scores and bias of imitation fidelity for speed and path length measures. These analyses provided group activation patterns for the different measures using a *p*-value of 0.001 uncorrected with an extent threshold of 38 voxels (following Monte-Carlo simulations by Slotnick et al., 2003), which left only the clusters that were considered significant at an FWE-corrected threshold of *p* < 0.05.

## Results

This pilot study revealed correlations between simple imitation with the handle and between-subject variations in complex imitation. Different measures of imitation were explored, to see how they would elicit differing activation patterns.

### Behavioral data

The average path length correlation “R” between model and participant was 0.89 (SD = 0.06). For path length divided by time, the average was 0.93 (SD = 0.05). In terms of error scores, the average path length error was 201.85 pixels (SD = 53.52), and the average speed error score was 39.86 s (SD = 14.46 s, including the RMSE outlier of 2 SD > mean). There was no significant correlation between the *R*, RMSE or *m*-scores, and age. The R and RMSE scores correlated non-significantly at *p* = −0.504. Correlations between *m* and *R* (*p* = 0.198) or RMSE (*p* = 0.410) were not significant. Participants showed particular difficulty identifying the pentagonal shape, resulting in wide variations in drawings. All participants except one failed to decrease their speed on par with the model, resulting in a rate of change “*m*”<1 (1 = same increase in speed for model and participant between all trials). The average motor bias “*m*” for speed was 0.804 (SD = 0.15). For path length “m”, performance was variable, with the rate of change both over and under 1 averaging at 0.987 (SD = 0.07).

### Functional data

**Figure 3:**
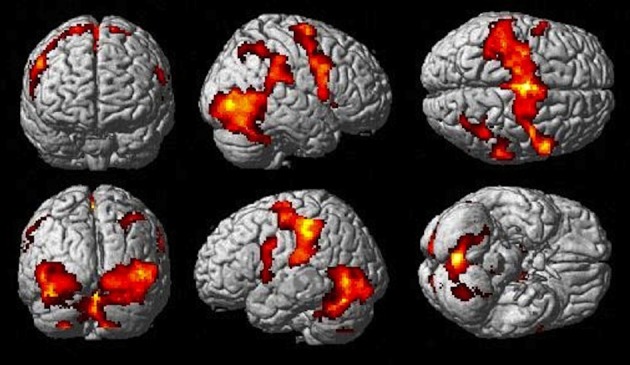
**Imitate BOLD contrast (*p* < 0.05 FWE-corr.)**.

The Observe group contrast (i.e., Watch-minus-Rest) revealed significant activation only in the visual cortex. The Imitate contrast (i.e., Move-minus-Rest) on the other hand (Figure 3) revealed activation predominantly in the bilateral cerebellum, but also in the left postcentral parietal lobe, inferior frontal gyrus and thalamus.

One participant was excluded from all BOLD analyses due to an unalterable shift in the functional MR-images and non-compliance in the “Move” handle-imitation condition.

### Correlates of imitation accuracy “R” with BOLD signal changes

**Figure 4:**
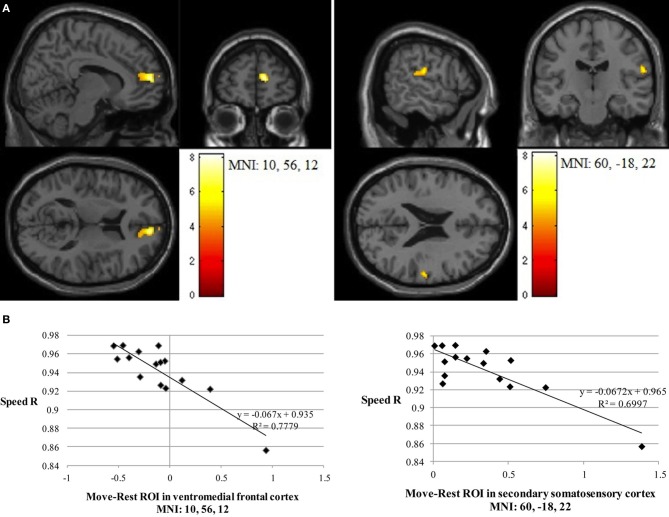
**(A)** The two significant clusters in the negative correlation between speed accuracy (R) and BOLD response in Imitate (*p* < 0.05 FWE-corr.). **(B)** Scatter-plots for both ROIs show how speed “R” correlates to BOLD signal across participants. Average BOLD response for each condition was calculated over a 5 mm sphere around the peak of the ROI, after which Rest was subtracted from Move for each participant to reflect differential activation during Imitate.

Path length correlated negatively with Imitate in the left supramarginal gyrus of the postcentral parietal lobe (MNI: −40, −22, 46; *Z* = 3.96, cluster size 46). A negative correlation between speed *R* and Imitate revealed activity in the right ventromedial frontal cortex (MNI: 10, 56, 12; *Z* = 4.77, cluster size 180) and the right secondary somatosensory cortex (MNI: 60, −18, 22; *Z* = 4.07, cluster size 120; both in Figure 4). Scatter-plots (Figure 4B) illustrate the nature of the whole-brain negative correlations by comparing speed *R* with average BOLD response in the Move minus the Rest condition for the two regions-of-interest (ROIs). There was a positive correlation between Observe and path length in the area of the right caudate (MNI: 22, −10, 28), although this correlation was only borderline significant (*Z* = 3.85, cluster size 40). There was no significant correlation between Observe and speed R.

### Correlates of BOLD response with behavioral measures of imitation bias (gradient *“m”*)

**Figure 5:**
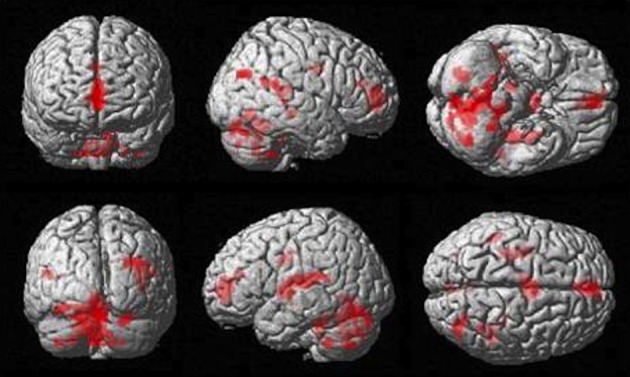
**Group activation found in the negative correlation between the BOLD Imitate contrast and rate of change in speed “*m*” (14 participants, *p* < 0.05 FWE-corr.)**.

The more accurately participants' speed matched that of the model, the less activity they showed during simple imitation in a range of areas shown in Figure 5 and Table 1. This relationship was strongest in the cerebellum but symmetrical clusters were also evident in the posterior insula and midline in ventro- and dorsal medial frontal cortex as well as posterior intra-parietal sulcus. Imitate did not correlate significantly with path length. However, path length *m* was positively correlated to Observe in the left superior frontal gyrus (MNI: −6, 64, −4; *Z* = 3.61, cluster size 71). There was no correlation between speed and Observe.

**Table 1:** **Locations, significance (at *p* < 0.05 FWE-corr.), and MNI coordinates for the negative group correlation between BOLD in Imitate and the speed bias (14 participants)**.

**Location**	**Cluster size**	***Z*-score**	***x***	***y***	***z***
l. Vermis	950	5.02	−2	−66	−6
r. Cerebellum	86	4.45	24	−72	−26
r. Anterior cingulate	197	4.15	2	−2	34
l. Cerebellum	140	4.14	−4	−34	−24
l. Insula	346	4	−30	−28	14
r. Cerebellum	95	3.99	14	−50	−50
r. Thalamus	52	3.92	14	−26	−8
r. Precuneus	120	3.86	32	−70	28
Medial frontal gyrus	336	3.8	0	52	−2
l. Fusiform gyrus	57	3.77	−38	−70	−16
r. Insula	145	3.7	30	−30	14

### Correlates of BOLD signal with mean error (RMSE) in complex imitation

**Figure 6:**
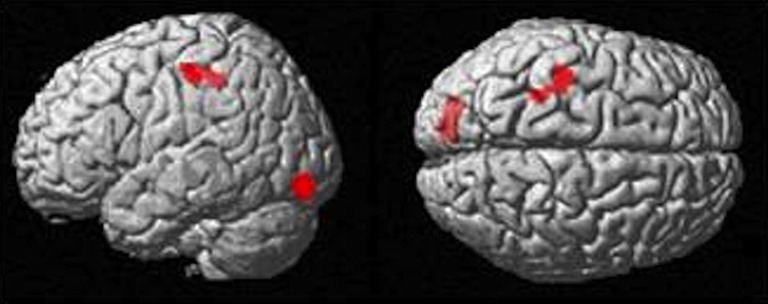
**Group BOLD response in positive correlation with speed RMSE data (*p* < 0.05 FWE-corr.)**.

The Imitate contrast did not correlate with path length. There was, however, a positive group correlation (after the removal of the RMSE outlier) between speed and Imitate in the left postcentral gyrus (specifically the somatosensory cortex, leading into the intra-parietal sulcus, with MNI: −38, −24, 50; *Z* = 4.20, cluster size 147), and in the visual cortex (MNI: −16, −86, 10; *Z* = 4.14, cluster size 229; both in Figure 6). There were no significant correlations between the Observe contrast and RMSE measures.

## Discussion

In this study we investigated if individual differences in brain activity during a very simple imitation task correlated with performance on a challenging behavioral imitation task for three different measures. We predicted that matching accuracy on a difficult task would correspond to activity during a simple action-perception matching task in the overall imitation system (Caspers et al., 2010), whereas bias would be under the control of more general motor control functions. Our hypothesis was partially confirmed for the imitation of speed. The strength of correlation “*R*” predicted BOLD signal in the somatosensory cortex in right anterior parietal lobe but also right ventromedial prefrontal cortex. The measure of bias (“*m*”) showed multiple associations with general motor control and attention functions in bilateral cerebellum, thalamus and the right precuneus but also bilateral posterior insula, left medial frontal cortex in two separate clusters; one anterior and the other posterior. RMSE identified a left somatosensory cortex correlation and visual cortex activation.

Before considering these specific associations any further, some discussion of the nature of the association is warranted. Firstly, our main objective was not to establish the neural substrate of imitation but to explore the sources of *variability* within a group of typical individuals. We do not claim that the brain areas identified are critical for manual imitation but rather, we suggest that these areas contribute to the accuracy and precision of manual imitation, particularly by mediating the dependence of motor output on sensory input such that differences in their function during imitation contribute to variability in imitation performance. A second important issue is that the nature of the two imitation tasks differed. Though both concerned manual imitation, the scanner task relied on selection of a goal-directed grasping action, whereas imitation using the touchscreen relied on drawing skill. This may be considered a limitation, but it means that only neurocognitive functions common to both tasks are likely to be identified, and therefore that any positive findings are more generalizable to other manual imitation tasks. Indeed our findings identified areas engaging the imitation system described by Caspers et al. (2010). Thirdly, in all cases where we found a relationship, this was negative, meaning that better imitation ability in the drawing task correlated with reduced BOLD signal from the brain areas identified in the scanner task. This means that the more skilled a person is at imitating, the less active these areas would be during a task as simple as the one used in our scanning experiment. This is supported by previous research on the effects of expertise (Vogt et al., 2007), assuming the areas concerned are adapted specifically to serve the function of imitation and therefore show greater activity for more demanding tasks. In terms of cross-modal feedback, a task experienced as “easy” by a skilled imitator would not require much sensitivity to feedback and so the most able imitators would show the least activation.

FMRI correlates of the accuracy measure (correlation) were largely confined to imitation-related activity in the ventromedial prefrontal cortex and anterior parietal cortex. The involvement of anterior parietal cortex was predicted as a key component of the imitation system but it is less obvious why the medial frontal cortex was implicated, as midline activation is associated with more abstract, social forms of imitation (Uddin et al., 2007). In a thorough meta-analysis of cingulate connectivity and function, Beckmann et al. (2009) found motor and memory-related functions to be associated with more posterior aspects of cingulate cortex, whereas the anterior aspect was associated with reward-functions. Ventral anterior cingulate has been associated with autism-control group differences in imitation (Williams et al., 2006), and Ingersoll et al. (2003) showed that successful imitation is related to reward-feedback, which is especially effective in a group generally considered poor at imitation. The fMRI paradigm used in this study meant that imitation required a correct selection of possible actions, which would likely generate activity in ventromedial frontal cortex. Therefore, an interpretation of our findings is that the degree to which simple imitation is experienced as rewarding predicts both the ability to imitate and sensitivity to feedback. The additional relation between midline frontal cortex and social cognition suggests that participants sensitive to social reward, i.e., motivated to perform the task they are asked to do, would experience the task as more rewarding. The data therefore leads us to hypothesize that if comparing a typical individual's imitation abilities with others, that person's sensitivity to feedback and capacity to learn to map this to an appropriate motor response will be the most important factors determining performance. While more attention might be required for imitation compared to observation, the absence of findings relating to the temporo-parietal junction indicates that biological motion perception, or theory of mind (Saxe, 2006; Mitchell, 2008), was not a predominant factor in the analyses. The visual cortex, however, was found to be significantly activated in the RMSE analysis, which Decety et al. (1997) found to be more active when attending to actions for purposes of imitation. The fact that this activation was not found in all analyses suggests only specific aspects of the task might be modulated by attention, with imitation as a whole comparable in visual activation to the observation condition.

Thalamus, intraparietal sulcus, insula, and cerebellum are all closely concerned with integrating multimodal sensory and motor feedback (Gallese et al., 2004; Dijkerman and de Haan, 2007). Models of motor control in motor imitation (Wolpert et al., 2003; Williams et al., 2007) suggest that visual information, whether from self or other, is fed into feedback systems, which provide cross-modal translation to inform motor planning functions. Correlations between BOLD activity and fidelity measures in these areas suggest that they may be important in mediating feedback sensitivity. Additionally, activation of the insulae, cerebella, and right thalamus in the group correlation with the bias measure suggests that innate motor bias can be functionally dissociated from sensory feedback by looking at a different measure of fidelity.

### Limitations and the future

Kinematics measured by a computer-drawing task, as a method of determining imitative ability, has only recently been developed and we emphasize the preliminary nature of this study which represents an initial exploration of the neural determinants of kinematic imitation ability. Our population was limited and consisted solely of males. It will be necessary to ascertain whether these findings extend to larger and different populations, including females and groups known to have difficulties with imitation tasks. We recognize that group comparison research will require an additional motor-execution condition, but posit that the homogeneity of the current participant group and overall task performance at ceiling level in the scanner task were enough to ensure that any possible differences in motor ability did not affect the results.

Future research will aim to test kinematic imitation ability in people with ASD, a heterogeneous group that has in the past shown inconsistent findings of an imitation and mirror neuron deficit (e.g., Press et al., 2010). Research using kinematics will allow us to see if a discrepancy in imitation skill between this group and neurotypicals can be accounted for by a deficit in action-perception matching or if the variability in imitation fidelity between individuals is driven by variable function in broader motor-control systems. The ability of the manual imitation task to objectively separate different kinematic measures will furthermore allow future research to determine whether there is an imitation deficit in ASD that is specific to temporal or spatial aspects. Objective and quantifiable measurements of imitation ability should be more sensitive to small group differences and performance can be compared to highly comparable non-imitation tasks. For example, in a recent study (Stewart et al., under review) imitation ability using the paradigm described in this study, was compared to performance on a highly comparable “ghost” condition where only the target movement and not the action was displayed. Similarly, other measures of motor ability can be derived from KAT or other kinematic methods, and it will be possible to further investigate the motor correlates of imitation ability in general or of group-differences between autism and neurotypical groups. These approaches will be useful in investigating whether a multiplicity of different motor problems could be contributing to the heterogeneity of ASD.

As mentioned before, the difference between tasks in and out of the scanner helps to reveal common neural substrates, yet also inevitably raises the question of how individual variability in performance will correspond to differences in BOLD signals if tasks are more similar. The next step in researching the relation between complex manual imitation and its neural substrates will be to run the objective imitation task in an fMRI environment. This requires the development of appropriate kinematic measures that can be collected in that environment. Only then can the imitation measures be applied to an ASD population and be able to truly compare brain activation between groups.

## Conclusion

Overall, this study has taken a novel approach to studying manual imitation fidelity and its neural correlates. We investigated the possibility of overlapping neural substrates between simple and challenging imitation tasks and the influence of between-subject variance on this overlap. Inside the scanner, the participants performed a simple imitation task requiring depression of a handle. To measure imitation skill, participants performed a separate imitation-drawing task using touch-screen software. Three different measures of performance on the complex imitation task were correlated with cortical activity during simple imitation. This provided evidence of increased activity in not only mirror neuron areas, but also areas that serve sensory feedback, sensorimotor integration, and reward-related learning, with increasing task demands. This means that activity in these areas is less for those people with better imitation ability. We conclude that imitation is a complex skill, and that the different components of imitation fidelity can be functionally separated to reveal how they influence error in variable but measureable ways.

### Conflict of interest statement

The authors declare that the research was conducted in the absence of any commercial or financial relationships that could be construed as a potential conflict of interest.
